# Role of the Soluble Receptor for Advanced Glycation End Products (sRAGE) as a Prognostic Factor for Mortality in Hemodialysis and Peritoneal Dialysis Patients

**DOI:** 10.1155/2018/1347432

**Published:** 2018-10-15

**Authors:** Elena Dozio, Federico Ambrogi, Massimo de Cal, Elena Vianello, Claudio Ronco, Massimiliano M. Corsi Romanelli

**Affiliations:** ^1^Department of Biomedical Sciences for Health, Laboratory of Clinical Pathology, Università degli Studi di Milano, Milan, Italy; ^2^I.R.C.C.S. Policlinico San Donato, San Donato Milanese, Milan, Italy; ^3^Department of Clinical Sciences and Community Health, Laboratory of Medical Statistics, Biometry and Epidemiology “G.A. Maccaro”, Università degli Studi di Milano, Milan, Italy; ^4^Department of Nephrology, Dialysis & Transplantation, San Bortolo Hospital, Vicenza, Italy; ^5^International Renal Research Institute Vicenza (IRRIV), San Bortolo Hospital, Vicenza, Italy; ^6^Service of Laboratory Medicine 1-Clinical Pathology, I.R.C.C.S. Policlinico San Donato, San Donato Milanese, Milan, Italy

## Abstract

End-stage renal disease patients on dialysis (CKD-G5D) have a high mortality rate due to cardiovascular diseases (CVD). In these patients, inflammation, oxidative stress, and uremia increase the production of glycation products (AGEs) which in turn accelerate CVD onset and progression. Recently, attention has been given to the soluble receptor for AGEs (sRAGE) as a marker of inflammation, oxidative stress, atherosclerosis, and heart failure in CKD-G5D. However, its association with patient outcomes is still under debate. Our aim is to explore whether sRAGE may be a predictor of mortality in CKD-G5D. We studied 123 CKD-G5D for 24 months. Of these patients, 56 were on hemodialysis (HD) and 67 on peritoneal dialysis (PD). Demographic, anthropometric, biochemical, and clinical data were recorded. sRAGE was quantified by enzyme-linked immunosorbent assay. sRAGE was a predictor of mortality at *2*-year follow-up. Each increase of 100 pg/mL in sRAGE levels was associated with an approximately 7% increased risk of mortality. Furthermore, in the entire study group, as well as in PD and HD patient subgroups, sRAGE was positively correlated with brain natriuretic peptide (BNP) levels. Mortality rates as well as sRAGE levels in patients who died did not differ between PD and HD patients. In conclusion, the positive association observed with BNP levels suggests a role for sRAGE as a prognostic factor for mortality in CKD-G5D patients displaying an active process of cardiac remodeling.

## 1. Introduction

End-stage renal disease (ESRD) patients on dialysis (CKD-G5D) have a high mortality rate due to cardiovascular diseases (CVD), infections, and malnutrition. In addition to traditional CVD risk factors, such as hypertension, diabetes mellitus (DM), and dyslipidemia, which are common in these patients, excessive oxidative stress and chronic inflammation may further increase cardiovascular morbidity and mortality [1, 2].

Products of glycation (AGEs) derive from glycation and oxidation of proteins, lipids, and nucleic acids. AGEs increase in conditions of hyperglycemia, oxidative stress, and uremia and in turn promote inflammation, reactive oxygen/nitrogen species production, apoptosis, and metabolic dysfunctions. In this way, AGEs may accelerate CVD onset and progression [3–5].

In the field of CVD, recent attention has been given to the receptor for AGEs (RAGE), a membrane receptor which mediates the damaging effects of these compounds. Besides the cell membrane form, RAGE also exists as a soluble molecule, sRAGE. This soluble receptor, by binding AGEs and other RAGE ligands in the circulation, prevents intracellular RAGE signaling and related proinflammatory effects [6]. In cardiometabolic diseases, low rather than high sRAGE levels can be observed, thus reinforcing the idea of a protective role for this molecule [7, 8]. However, DM and/or ESRD patients, groups with a high risk of CVD, show increased sRAGE levels [9–11]. This phenomenon may be strongly related to inflammation and oxidative stress which promote the expression of RAGE on the cell membrane and its cleavage by metalloproteinases (MMPs) [12–14]. Moreover, in ESRD, the accumulation of sRAGE is also contributed by reduced renal function [15].

Despite a link between levels of sRAGE with parameters of inflammation, oxidative stress, atherosclerosis, and heart failure (HF) in CKD-G5D [16–20], prior studies failed to observe a strong association with patient outcomes [21–24]. However, there are a limited number of studies in this field, and they are difficult to compare due to differences in study design and patient features. In particular, most studies have been focused on accompanying atherosclerosis and vascular calcification as a part of ESRD [21–24].

Since we have recently described a role for sRAGE as a marker of cardiac remodeling in a group of CKD-G5D patients [8], we evaluated the prognostic role of sRAGE in this well-characterized cohort which include both hemodialysis (HD) and peritoneal dialysis (PD) patients. We hypothesized that in those patients with active cardiac remodeling, elevations in sRAGE may have a prognostic significance, and moreover, sRAGE may be a future target for beneficial therapies.

## 2. Materials and Methods

### 2.1. Source Population

In this prospective, observational cohort study, we analyzed 123 patients on various dialysis modalities. Of these, 56 were on HD and 67 on PD. To be enrolled in the study, patients had to (1) be on HD or PD treatment for at least 3 months, (2) agree to participate in the study with written inform consent, and (3) be ≥18 years. Patients with missing or incomplete clinical history, inability to provide consent, and hepatic encephalopathy were excluded. The observation period was 24 months. During the follow-up period, 23 patients died. This study was performed in accordance with the Declaration of Helsinki, as revised in 2013. The protocol was approved by the Ethics Committee of San Bortolo Hospital (N.41/14).

### 2.2. Clinical and Laboratory Parameters

Demographic, anthropometric, biochemical, and clinical data (i.e., age, gender, smoking status, alcohol consumption, hypertension, DM, and CVD) were collected. Blood samples were collected during outpatient visits for PD patients or prior to hemodialysis treatment after the long interdialytic interval. Samples for nonroutine assays were collected in EDTA. Plasma was separated by centrifugation, immediately frozen at −80°C, and stored until measurements. Routine biochemical parameters were assessed as previously described [20]. sRAGE was measured by a commercial human ELISA kit (R&D Systems, Minneapolis, MN, USA) according to the manufacturer's instructions. The minimum detectable concentrations ranged from 1.23 to 16.14 pg/mL. The maximum intra- and interassay coefficients of variations were, respectively, 4.8% and 8.3%. Samples have been assayed in duplicate. The GloMax®-Multi Microplate Multimode Reader was used for photometric measurements (Promega, Milan, Italy).

### 2.3. Statistical Analysis

Quantitative variables are expressed as mean with standard deviation (SD) and median with interquartile range. Qualitative variables are summarized as numbers and percentages. The normality of data distribution was assessed by the Kolmogorov-Smirnoff test. Comparison between two groups was performed by Mann-Whitney test for continuous variables and *χ*^2^ test for nominal variables. The potential univariate association between sRAGE and the selected variables was performed with Pearson's (for normal-distributed data) or Spearman's (for non-normal-distributed data) correlation tests.

The association of sRAGE with mortality in HD and PD patients was evaluated with logistic regression considering possible nonlinear effects using restricted cubic splines with 3 knots. The association was then adjusted considering other possible confounding factors taking into account the number of events. To select possible confounding variables among those available (age, gender, body mass index (BMI), smoking, alcohol, DM, hypertension, CVD, total protein, urea, triglycerides, LDL-cholesterol, HDL-cholesterol, brain natriuretic peptide (BNP), and glucose), a LASSO penalized logistic regression was used. Regression analyses are represented as OD and 95% CI. Differences were considered significant at *p* < 0.05 (95% CI).

## 3. Results

### 3.1. Baseline Characteristics of Patients

The baseline characteristics of the patients included in the study are presented in Table 1. There was no evidence of difference in the mean age of HD and PD groups as well in the existing comorbid conditions. Concerning anthropometric and biochemical parameters, it is noteworthy that PD patients had higher body BMI and increased levels of alanine aminotransferase (ALT), aspartate aminotransferase (AST), LDL-cholesterol, total cholesterol, and urea levels in comparison with HD patients. Moreover, PD patients had lower uric acid, BNP, and albumin levels. Also, sRAGE concentration was lower in PD patients. No significant difference was observed in total protein and C-reactive protein (CRP) levels. The number of patients who died during the follow-up period was 23. Of these, 10 were HD and 13 were PD patients with similar percentage of death between the two groups. There was no significant difference in the levels of sRAGE between HD and PD patients who died (4597.38 ± 930.18 pg/mL vs. 3480.86 ± 1538.62 pg/mL; *p* = 0.07).

### 3.2. Univariate Association of sRAGE with Clinical Parameters

The results of the correlation analyses are shown in Table 2. In the whole group of patients, sRAGE was positively correlated with BNP (*r* = 0.353, *p* < 0.001) and negatively with LDL-cholesterol (*r* = −0.365, *p* < 0.001) and total cholesterol (*r* = −0.339, *p* < 0.001) levels. Similarly, in the HD group, sRAGE was positively correlated with BNP (*r* = 0.281, *p* < 0.05) and negatively with LDL-cholesterol (*r* = −0.433, *p* < 0.001) and total cholesterol (*r* = −0.456, *p* < 0.001). In the PD group, we observed a positive correlation of sRAGE with BNP (*r* = 0.334, *p* < 0.01) and a negative correlation with CRP (*r* = −0.241, *p* < 0.05) levels.

### 3.3. Association of sRAGE Levels with Mortality

There was no evidence of nonlinear effect for the association of sRAGE with mortality (*p* = 0.33); therefore, only the linear effect was retained. According to the LASSO penalized logistic regression, the following variables were selected and then utilized for the multivariable regression model: age, BNP, glucose, HDL-cholesterol, smoking, and sRAGE. In particular, three regression models are reported (Table 3): model 1 with only sRAGE (univariate); model 2 adjusted for age, gender, glucose, and HDL-cholesterol; and model 3 adjusted also for BNP. sRAGE was associated with mortality in models 1 and 2 (*p* = 0.016 and *p* = 0.006, respectively). Considering model 2, a difference of 100 pg/mL in the value of sRAGE concentrations corresponds to a difference of about 7% in the probability of death. In model 3, which further includes BNP as the adjustment factor, the statistical significance was lost (*p* = 0.096) (Table 3). We also observed an association between BNP and mortality in these patients with an odds ratio (OR) of 1.004 (95% confidence interval (CI): 1.001–1.008, *p* = 0.011). The association lost significance after adjusting for age, gender, glucose, HDL-cholesterol, and sRAGE (OR: 1.004; 95% CI: 0.999–1.009; *p* = 0.080).

## 4. Discussion

Our findings suggest for the first time a potential role for sRAGE as a prognostic factor for mortality at *2*-*year* follow-up in a group of CKD-G5D including both HD and PD patients. In detail, an increase of 100 pg/mL in sRAGE levels was associated with a significant increase in the risk of mortality of about 7%.

Moreover, although the concentration of sRAGE was higher in the HD than in the PD group, we observed that the rate of mortality as well as sRAGE levels in patients who died did not differ between the two types of dialysis modalities.

The role of sRAGE as a predictor of mortality in patients on dialysis is still under debate. The few studies that have been performed in this field up until now failed to observe any association [22, 24–26]. Only a single study observed a trend, although not statistically significant, towards increased mortality with higher rather than lower sRAGE levels [25]. While not completely clear, the reasons for such differences between our and other studies may relate to differences in the clinical characteristics of the patients enrolled.

In our patients, we observed an increase in BNP concentrations and a strong positive correlation with sRAGE levels. This result may relate to the finding that both BNP and NT-pro-BNP have been previously described to be elevated in CKD-G5D patients, and it is well known that they are strongly associated with left ventricular hypertrophy and systolic dysfunction and have a predictive potential for HF and mortality [27–30]. Most of the previous studies on sRAGE and mortality in CKD-G5D have been performed focusing on atherosclerosis and vascular calcification as the main CVD outcomes, while they did not explore specific parameters of cardiac remodeling and HF as well as their association with sRAGE [22, 24, 26]. Our study is the first one which highlights the existence of an increased mortality rate with higher sRAGE levels in patients with a greater increase in BNP, thus suggesting a potential role for sRAGE as a predictor of mortality most specifically in patients with a significant deterioration of heart function. To be noted, according to the LASSO penalized logistic regression, sRAGE and BNP were just two of the markers that were selected as potential variables related to mortality in our patients. Moreover, when BNP was included as the adjustment factor in the regression model, sRAGE lost the statistical significance as a predictor of mortality. These data seem to suggest that sRAGE and BNP could be related and could reflect the same outcome, but we cannot exclude that the lack of significance may be also due to a limited number of events observed in our population. Our hypothesis seems to be strongly supported by results obtained in other types of patients in which increasing sRAGE levels emerged as a marker of worsening heart function and mortality [31–35]. These studies, in fact, indicated the existence of an association between sRAGE and the severity of HF, whereby sRAGE levels are increased in patients with higher BNP levels. Additional studies in larger cohorts of patients and the inclusion of echocardiographic parameters could help to confirm these results.

A novel finding of our study is also the observation that sRAGE levels are higher in HD than in PD patients. Such a comparison has been previously performed only in the study by Isoyama et al. [25]. These authors observed no difference between the two groups not only in sRAGE levels but also in the other clinical parameters evaluated. The reasons why the levels of sRAGE are different in HD and PD patients could be related to the way the two treatments retain or remove the solutes and the presence of a residual renal function that probably confers some advantages to PD patients. However, it is also possible that specific clinical factors, including but not limited to body composition, nutritional status, inflammatory, and prooxidant factors, may affect the circulating molecules [36–38]. More studies are needed in this regard.

Besides being a biomarker, sRAGE is a bioactive molecule with important physiological and pathological functions. The putative role of membrane RAGE activation and sRAGE circulating levels in cardiovascular complications of CKD patients are still very controversial. Previous studies indicated that AGEs levels are higher in CKD patients and, by interacting with membrane RAGE, they may activate intracellular signaling pathways that lead to oxidative stress, inflammation, vascular stiffness, atherosclerosis, tissue remodeling, and fibrosis [39–41]. Blockage of RAGE has been suggested as one potential protective mechanism against AGE-induced cardiovascular complications and sRAGE may just work in this way by linking to AGEs and preventing their interaction with RAGE [6, 40, 42]. These observations seem to be in contradiction with our findings that showed an increased mortality associated with the upregulation of sRAGE levels. In this regard, it is important to recall that sRAGE is a circulating pool composed of two different forms, namely esRAGE, the endogenous secretory form, and cRAGE that derives from proteolytic cleavage of the membrane-bound molecule, and that they can have different roles. Among these forms, esRAGE seems to be the real decoy receptor with important protective functions, whereas cRAGE appears more as a surrogate marker of inflammation. Previous observations indicated that inflammation- and oxidative stress-related conditions are associated with a decrease in esRAGE levels promoted by RAGE activation and an increased cleavage of the surface RAGE induced by MMPs and other enzymes [12–14, 43–45]. The quantification of the two forms that form the circulating pool could probably help to shed more light on the real role of sRAGE in different pathological conditions. Unfortunately, we have not determined which type of sRAGE is increased in our patients. However, based on previous results, we expect to observe a major contribution by cRAGE. Accordingly, it is possible that the increased levels of sRAGE observed in HD patients compared to PD may be due partly to the presence of residual renal function in PD and partly to the increased inflammation and oxidative stress described in HD patients, two conditions that may increase cRAGE production [2, 46]. Noteworthy also is our observation of an inverse correlation between sRAGE and CRP levels in PD but not in HD patients. This data could thus be interpreted as a failed protective response of sRAGE in HD probably due to the increased production of cRAGE and the activation of a loop which further promotes inflammation, RAGE cleavage, and esRAGE downregulation. However, the real biological role of the different sRAGE forms in these patients remains to be elucidated.

Several limitations of this study should be acknowledged, starting with the number of patients included and the follow-up limited to 2 years that did not allow us to observe more events. Moreover, the availability of some additional clinical parameters, such as echocardiography data on heart function, inflammatory biomarkers, and sRAGE isoforms, would allow us to obtain a more unbiased estimation of the relations observed and also to confirm the inflammation milieu previously described in HD [2, 46].

## 5. Conclusions

In summary, our results revealed for the first time that in patients with active cardiac remodeling, elevations in sRAGE may have a prognostic significance. Although we cannot conclude whether sRAGE is a predictor of HF, its quantifications seem to be as a useful supportive tool that could help to identify high-risk patients. Whether sRAGE might also be a future target for beneficial therapies in these patients needs to be explored in the future.

## Figures and Tables

**Table 1:** Baseline characteristics of patients included in the study.

	All patients (*n* = 123)	HD (*n* = 56)	PD (*n* = 67)	*p*
*Clinical parameters*				
Age (years)	63.71 ± 12.98; 65.33 (53.46–73.18)	65.47 ± 14.25; 67.93 (55.18–76.14)	62.23 ± 11.72; 63.68 (52.90–71.23)	0.098
Male gender (*n*, %)	87, 70.73%	40, 71.43%	47, 70.15%	1.00
BMI (kg/m^2^)	26.64 ± 5.61; 26.10 (22.12–30.43)	25.65 ± 6.55; 22.98 (21.30–28.27)	27.36 ± 4.73; 27.20 (23.60–30.90)	**0.021**
Diabetes (*n*, %)	31, 25.20	17, 30.36%	14, 20.89%	0.298
Smoking (*n*, %)	15, 12.20%	6, 10.71%	9, 13.43%	0.784
Alcohol consumption (*n*, %)	4, 3.25	2, 3.57%	2, 2.98%	1.00
Hypertension (*n*, %)	75, 60.98	35, 62.5%	40, 59.70%	0.853
Cardiovascular diseases (*n*, %)	40, 32.52	20, 35.71%	20, 29.85%	0.564
*Biochemical parameters*				
Albumin [g/dL]	3.14 ± 0.34; 3.10 (2.90–3.40)	3.22 ± 0.35; 3.20 (3.00–3.48)	3.08 ± 0.33; 3.10 (2.80–3.30)	**0.033**
ALT [UI/l]	21.51 ± 11.78; 18.00 (14.00–25.00)	19.27 ± 11.01; 16.00 (13.00–23.75)	23.39 ± 12.16; 21.00 (16.00–26.00)	**0.010**
AST [UI/l]	12.25 ± 9.50; 11.00 (6.00–16.00)	10.29 ± 6.50; 9.00 (5.00–15.50)	13.90 ± 11.21; 12.00 (7.00–17.00)	**0.036**
BNP [pg/mL]	10333.00 ± 12240.00; 3662.00 (1613.00–18857.00)	14182.00 ± 13084.00; 9335.00 (2214.00–26815.00)	7115.00 ± 10546.00; 2500.00 (1049.00–6541.00)	**<0.001**
Creatinine [mg/dL]	9.26 ± 2.92; 9.20 (7.40–10.60)	9.20 ± 2.86; 9.05 (7.40–10.23)	9.32 ± 2.98; 9.50 (7.10–11.50)	0.525
CRP [mg/L]	1.06 ± 1.87; 0.40 (0.29–1.08)	1.30 ± 2.40; 0.54 (0.29–1.11)	0.86 ± 1.25; 0.37 (0.29–0.98)	0.280
Glucose [mg/dL]	112.90 ± 34.17; 99.00 (89.00–125.00)	114.00 ± 31.89; 102.50 (89.50–125.00)	111.90 ± 36.17; 98.00 (88.00–125.00)	0.486
LDL-cholesterol [mg/dL]	79.09 ± 32.96; 74.00 (55.00–101.30)	67.49 ± 28.67; 65.00 (48.00–79.00)	88.61 ± 33.38; 88.00 (62.00–110.00)	**<0.001**
HDL-cholesterol [mg/dL]	48.13 ± 14.42; 44.00 (38.00–54.50)	44.00 (40.00–54.00)	48.48 ± 15.60; 44.00 (36.00–57.00)	0.797
Total cholesterol [mg/dL]	157.70 ± 41.20; 154.00 (127.00–182.00)	142.20 ± 34.54; 139.50 (119.80–162.80)	170.60 ± 42.06; 164.00 (135.00–196.00)	**< 0.0001**
Total protein [g/dL]	6.99 ± 0.57; 7.00 (6.60–7.40)	7.06 ± 0.51; 7.05 (6.60–7.40)	6.92 ± 0.61; 6.90 (6.50–7.30)	0.132
Triglycerides [mg/dL]	155.50 ± 93.97; 132.00 (90.00–196.30)	142.10 ± 80.92; 126.00 (82.00–176.00)	166.40 ± 102.80; 140.00 (94.00–203.00)	0.144
Urea [mg/dL]	123.40 ± 37.05; 118.00 (98.00–145.00)	114.90 ± 38.80; 116.50 (91.50–137.00)	130.50 ± 34.21; 127.00 (111.00–154.00)	**0.019**
Uric acid [mg/dL]	5.67 ± 1.30;5.70 (4.60–6.40)	5.98 ± 1.34;5.90 (5.13–6.60)	5.40 ± 1.21;5.40 (4.40–6.20)	**0.014**
Deaths (*n*, %)	23, 18.70%	10, 17.86%	13, 19.40%	1.00
sRAGE [pg/mL]	3197.00 ± 1356.00; 3071.00 (2167.00–4299)	3613.00 ± 1395.00; 3692.00 (2393.00–4754.00)	2849.00 ± 1227.00; 2665.00 (1955.00–3890.00)	**< 0.0001**

Data are expressed as mean ± SD and median (25th–75th percentiles) or number and proportions. ALT, alanine aminotransferase; AST, aspartate aminotransferase; BMI, body mass index; BNP, brain natriuretic peptide; CRP, C-reactive protein; HD, hemodyalisis; PD, peritoneal dialysis; sRAGE, soluble receptor for advanced glycation end products. Comparison between groups was performed by Mann-Whitney *U*-test or Fisher exact test. *p* values less than 0.05 are indicated in bold.

**Table 2:** Correlations of sRAGE with baseline characteristics of patients included in the study analyzed as a whole group and after classification according to the dialytic treatment.

sRAGE	All patients	HD	PD
	*r*	*r*	*r*
Age (years)	0.073°	0.031	0.001°
Albumin [g/dL]	−0.071°	−0.108	−0.134
ALT [UI/l]	−0.029°	0.064°	0.094°
AST [UI/l]	−0.077°	−0.163°	0.135°
BMI (kg/m^2^)	−0.010°	0.146°	−0.037
BNP [pg/mL]	**0.353** ^∗∗∗^ **°**	**0.281** ^∗^ **°**	**0.334** ^∗∗^ **°**
Creatinine [mg/dL]	0.077	0.060°	0.067
CRP [mg/L]	0.006°	0.156°	**−0.241** ^∗^ **°**
Glucose [mg/dL]	−0.049°	−0.081°	−0.075°
HDL-cholesterol [mg/dL]	−0.019°	−0.200°	0.113°
LDL-cholesterol [mg/dL]	**−0.365** ^∗∗∗^	**−0.433** ^∗∗∗^	−0.210
Total cholesterol [mg/dL]	**−0.339** ^∗∗∗^	**−0.456** ^∗∗∗^	−0.130
Total protein [g/dL]	−0.056	−0.133°	−0.070
Triglycerides [mg/dL]	−0.127°	−0.111°	−0.069°
Uric acid [mg/dL]	0.087	0.115	−0.067
Urea [mg/dL]	−0.115	0.067	−0.196

ALT, alanine aminotransferase; AST, aspartate aminotransferase; BMI, body mass index; BNP, brain natriuretic peptide; CRP, C-reactive protein; HD, hemodyalisis; PD, peritoneal dialysis; sRAGE, soluble receptor for advanced glycation end products. Correlations were evaluated with Spearman's (°) or Pearson's correlation coefficient, as appropriate. ^∗^*p* < 0.05; ^∗∗^*p* < 0.01; ^∗∗∗^*p* < 0.0001.

**Table 3:** Adjusted odds ratio for overall mortality according to sRAGE levels.

Model	OR (95% CI)	*p* value
(1) Univariate	1.044 (1.009–1.083)	**0.016**
(2) Adjusted for age, gender, glucose, and HDL-cholesterol	1.067 (1.021–1.122)	**0.006**
(3) Adjusted for age, gender, glucose, HDL-cholesterol, and BNP	1.042 (0.994–1.096)	0.096

OR, odds ratio; CI, confidence interval; sRAGE, soluble receptor for advanced glycation end products. Odds ratios are for increment of 100 in the value of sRAGE. *p* values less than 0.05 are indicated in bold.

## Data Availability

The data used to support the findings of this study are available from the corresponding author upon request.
